# The Real-World Impact of Vaccination on COVID-19 Cases During Europe’s Fourth Wave

**DOI:** 10.3389/ijph.2022.1604793

**Published:** 2022-07-05

**Authors:** Vageesh Jain, Aimee Serisier, Paula Lorgelly

**Affiliations:** ^1^ Institute for Global Health, UCL, London, United Kingdom; ^2^ Mid and South Essex NHS Trust, Essex, United Kingdom; ^3^ Institute for Epidemiology & Health Care, UCL, London, United Kingdom

**Keywords:** COVID-19, Europe, public health restrictions, Econometrics, vaccine effectiveness

## Abstract

**Objectives:** Disease control is important to limit the social, economic and health effects of COVID-19 and reduce the risk of novel variants emerging. Evidence suggests vaccines are less effective against the Omicron variant, but their impact on disease control is unclear.

**Methods:** We used a longitudinal fixed effects Poisson regression model to assess the impact of vaccination on COVID-19 case rates across 32 countries in Europe from 13th October to 01st January 2022. We controlled for country and time fixed effects and the severity of public health restrictions.

**Results:** Full vaccination coverage increased by 4.2%, leading to a 54% reduction in case rates across Europe (*p* < 0.001). This protection decreased over time but remained significant at 5 weeks after the detection of Omicron. Mean booster vaccination rates increased from 2.71% to 24.5% but provided no significant additional benefit. For every one-unit increase in the severity of public health restrictions, case rates fell by a further 2% (*p* = 0.019).

**Conclusion:** Full vaccination significantly limited the spread of COVID-19 and blunted the impact of the Omicron variant, despite becoming less useful over time.

## Introduction

Europe’s latest (and fourth) wave of COVID-19 started in mid-October 2021 [1], with several countries putting in further restrictions to limit the spread of disease and pressure on health systems. By early November, Europe was once again “at the epicentre” of the Covid pandemic, according to the World Health Organization (WHO) [2]. WHO Europe head Hans Kluge attributed insufficient vaccine uptake, which had stalled in several European countries over the Autumn months, and a relaxation of public health measures for rising infections in the region [2]. Given the relatively early use of COVID-19 vaccines in Europe, Dr Mike Ryan (Executive Director of the WHO Health Emergencies Programme) said Europe’s experience was a “warning shot for the world” [3].

The Omicron variant has intensified the spread of COVID-19 in Europe. The variant was first reported to the WHO by South Africa on 24th November 2021 [4]. Just over 1 week later on the 2nd December 2021, 375 cases with the variant were recorded across at least 30 countries [5]. Europe’s first case of Omicron was confirmed in Belgium on 26th November, and traced back to at least 22nd November [6]. With an expected lag between symptom onset and testing, experts believe that Omicron may have been first present in Europe prior to late November [7]. By 16th December, Omicron cases had been reported in all but three countries in Europe [8].

The WHO has categorized Omicron as a variant of concern, stating that early evidence suggests it may carry a higher re-infection risk, and be more transmissible [9]. In laboratory-based studies it is less susceptible to one or two doses of existing vaccines [10], but Pfizer recently reported that a third (booster) dose of BNT162b2 increases neutralizing antibody titres by 25-fold compared to two doses [11]. A small UK test-negative case control pre-print study of 581 symptomatic Omicron cases found vaccine effectiveness to be significantly lower compared to the Delta variant [12]. From 2 weeks after a booster, vaccine effectiveness against symptomatic disease increased to 70%–75% and boosters are thought to be largely effective in limiting severe disease.

On average, 62% of the European population were fully vaccinated by 13th January 2022 [13], but vaccine-derived immunity is known to wane over time [14]. For Omicron, booster effectiveness has been shown to wane over time, falling by 15%–25% after 10 weeks [15]. Several European countries have shortened or sped up booster rollouts in response to Omicron [16], but it is unclear what impact this has had. In addition, some governments have tightened public health restrictions over the Christmas period [17], with a similarly unclear impact upon disease control. Although data suggest Omicron is less severe than previous variants [18, 19], disease control is important to limit the widespread economic and social impacts of illness and isolation, and to reduce the chance of future more harmful COVID-19 variants.

This longitudinal study evaluates the protective impact of vaccination on COVID-19 disease control across Europe during the fourth wave of infections, from mid-October 2021 to January 2022. This will help policymakers understand how effective vaccines, including boosters, have been in controlling the spread of COVID-19 and whether this changed since the detection of the Omicron variant.

## Methods

### Study Design

We used a longitudinal fixed effects Poisson regression model to assess the impact of vaccination on COVID-19 case rates in Europe from 13th October to 1st January 2022. All included countries were part of the European Economic Area (EEA), with the addition of the UK and Switzerland. This study period allowed us to evaluate the impact of vaccination during the initial phase of a new wave of infections [1]. We did not include data from after 2022 to limit the chance of misattributing a change in case rates due to the rapid spread of the Omicron variant and resulting natural immunity [20], to the impact of vaccines, and due to the limited accuracy of routinely recorded cases during periods of high COVID-19 incidence. Fixed-effects models exploit the longitudinal nature of the data by relating within-country changes in case rates to within-country changes in time. They are a useful method for estimating causal effects because they account for any unobserved confounders that may vary across countries but are constant and have stable effects over time. This means we can account for important time-invariant differences across countries such as demographics and time-variant differences affecting all countries equally such as season. We also adjusted for the severity of public health restrictions, which vary across both time and country. We evaluate the causal impact of an increase in vaccination (i.e., primary course/full and booster) coverage on COVID-19 case rates in Europe.

### Data Sources

A longitudinal panel was constructed which included 27 European Union countries, 3 EEA countries, and the UK and Switzerland. The panel contained data on new daily COVID-19 cases per million population at three and four-day intervals (i.e., two dates per week), from 13th October to 1st January 2022 [13]. Data on COVID-19 vaccine coverage and the strength of government restrictions (measured through the Oxford COVID-19 Government Response Tracker (CGRT) Stringency Index) [13] were recorded for each country and at each time point. The Stringency Index combines nine different indicators: school closures, workplace closures, cancellation of public events, restrictions on public gatherings, closures of public transport, stay-at-home requirements, public information campaigns, restrictions on internal movements, and international travel controls. Where data were not available, they were treated as missing.

### Statistical Analysis

All analyses were conducted using STATA 14 [21]. We assessed changes in COVID-19 case rates, vaccination rates, and public health restrictions, across all countries over time using line graphs. Histograms were constructed showing a right-skewed distribution for COVID-19 case rates (Supplementary File S1).

We fit two longitudinal fixed effects Poisson regression models to investigate the impact of full vaccination (i.e., two doses for most vaccines) and booster vaccination (i.e., following a full primary course) on COVID-19 cases per million population, over the study period. We controlled for country and time fixed effects as well as the severity of public health restrictions. Both models were implemented through the *xtpoisson* command in STATA. Vaccination rates and the severity of public health restrictions were lagged by two time periods (representing 1 week), to allow time for changes to impact on cases. In all regression analyses, *p*-values of <0.05 and 95% confidence intervals excluding a value of one were considered statistically significant. Clustered standard errors were used to account for heteroskedasticity, autocorrelation and cross-sectional dependence. Because this is a fixed-effect longitudinal regression, we only evaluate changes *within* countries (i.e., changes in *within-*country case rates and *within*-country vaccination coverage). Between-country effects (i.e., differences between countries) are not estimated.

The regression model used in the study can be shown as:

$$log\left(y_{it}\right)=\alpha_{i}+\beta_{1}x_{1\;it}+\beta_{k}x_{k\;it}+\beta_{2}Z_{i}+\varepsilon_{it}$$

Where: *t* refers to the time period (i.e., three to 4 day intervals from 13th October to 01st January 2022) and *i* refers to an individual country; *y*_*it*_ is the number of new daily COVID-19 cases per million population in country *i* at time period *t*;

*α*_*i*_ is the fixed effect (country-level time-invariant effect) error term; *β*_*1*_ *x*_*1 it*_ is the coverage (from 0 to 100%) of full vaccination in country *i* in time period *t* with a coefficient of *β*_*1*_; *β*_*k*_ *x*_*k it*_ refers to each covariate (*k)* for country *i* in year *t* with coefficient *β*_*k*_ (i.e., observed time-variant heterogeneities across countries including severity of public health restrictions)

*β*_*2*_ Z_*i*_ refers to unobserved time-invariant heterogeneities across countries with a coefficient of *β*_*2*_ *;*

*ε*_*it*_ is the idiosyncratic error term for country *i* in year *t*; The parameters were exponentiated to aid interpretation:

$$y_{it}=exp\left(\alpha_{i}+\beta_{1}x_{1\;it}+\beta_{k}x_{k\;it}+\beta_{2}t+\varepsilon_{it}\right)$$

The exponentiated coefficients of traditional (count data) Poisson models are interpreted as incident rate ratios. Using a rate as our outcome measure, we exponentiate the regression outputs to obtain rate ratios (RR). The exponentiated coefficient *β*_*1*_ is the estimated ratio change of the COVID-19 case rate for every 1% increase in vaccination and is our main outcome of interest.

To evaluate the average marginal effect (AME) of vaccination rates for each time period (i.e., to understand whether vaccines became less effective over time), we constructed two further fixed effects Poisson regression models, each with an interaction term between time period and vaccination as well as the covariates used in previous models. We then used the *marginsplot* command in STATA to create graphs showing how the relationship between vaccination rates and predicted case rates changed over time, accounting for other variables in the model. The AME is calculated by first estimating the partial derivative of vaccination on case rates, using the observed values for the other covariates, and then estimating the average of first-order derivatives over the entire population.

## Results

**FIGURE 1:**
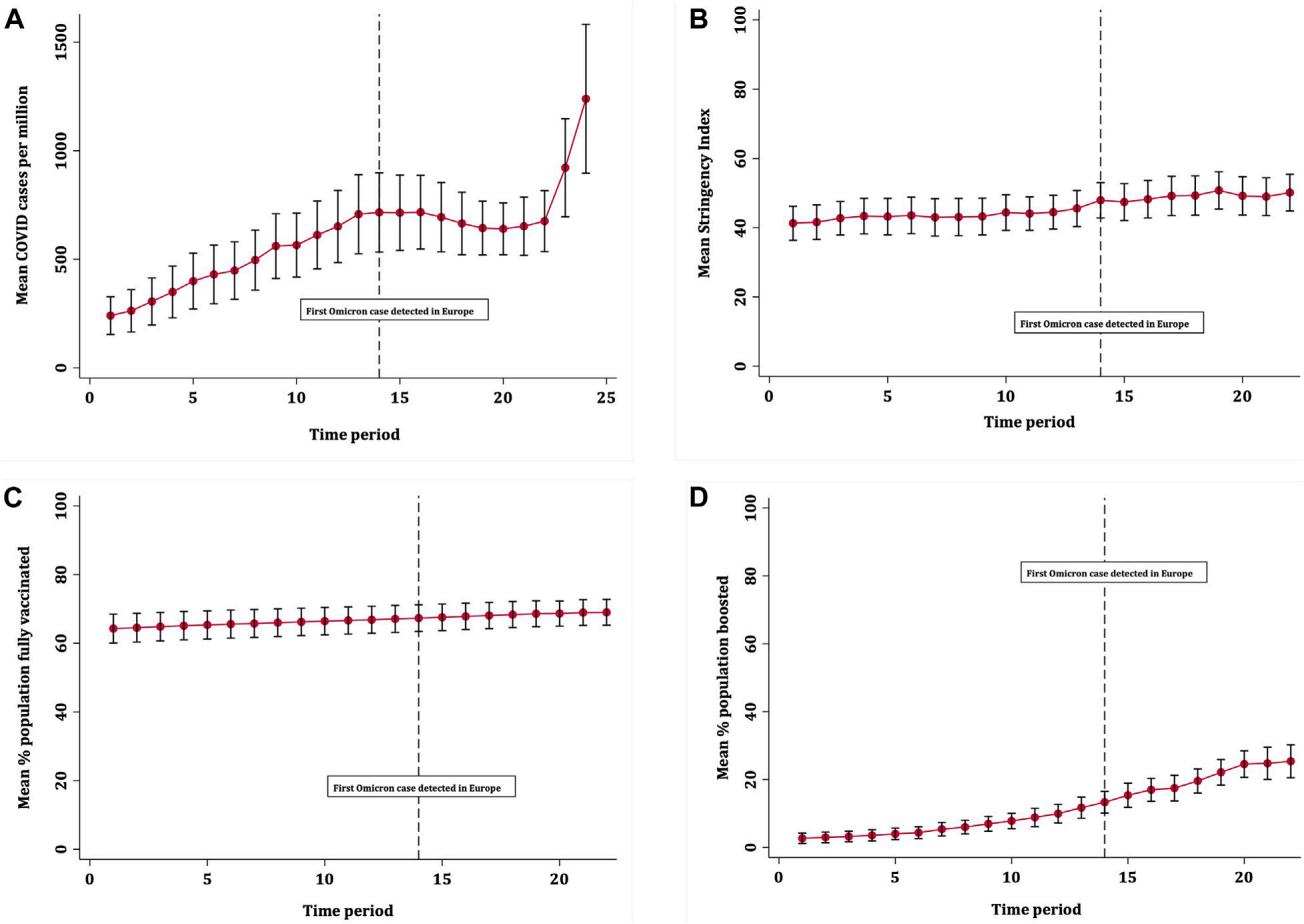
Trends in mean **(A)** COVID-19 case rates; **(B)** Stringency Index; **(C)** full vaccination rates and **(D)** booster vaccination rates, with 95% confidence intervals (Europe, 13th October 2021–01st January 2022).

**FIGURE 2:**
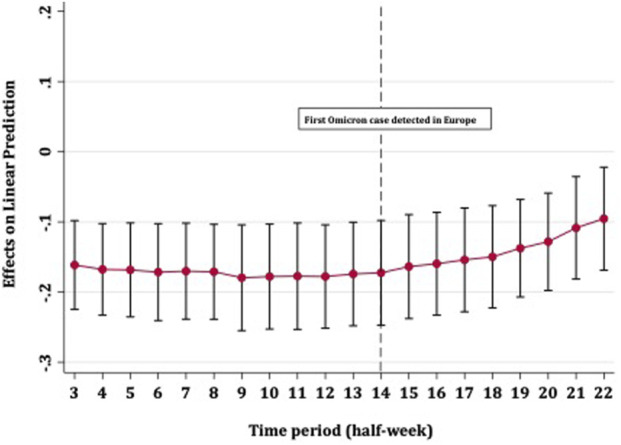
Average marginal effects of full vaccination on COVID-19 case rates over time with 95% confidence intervals (Europe, 13th October 2021–01st January 2022).

**FIGURE 3:**
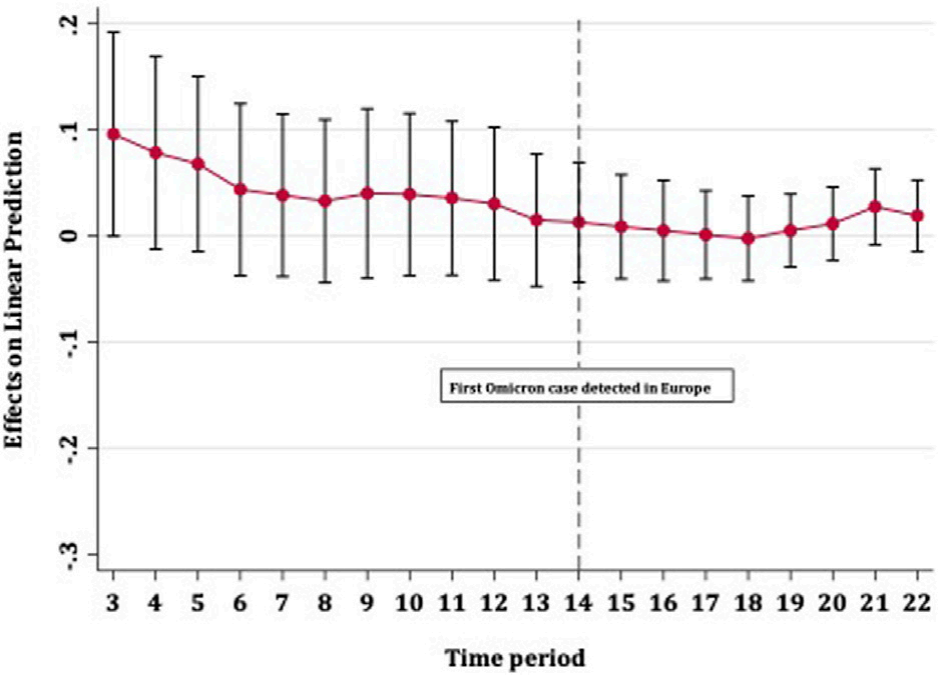
Average marginal effects of booster vaccination on COVID-19 case rates over time with 95% confidence intervals (Europe, 13th October 2021–01st January 2022).

Across 32 European countries and over the study period, there were 5,228,397 recorded COVID-19 cases. The mean COVID-19 case rate was 596 per million (SD 487), ranging from 240 per million at the start of the study period to 1239 per million by the end. Figure 1 shows that average COVID-19 case rates increased steadily over time until plateauing after time period 13 (24th November), around the time the first Omicron case was detected in Europe. Case rates then remained similar until the start of a drastic increase from 29th December 2021, although the confidence intervals for the last two time periods were wide due to more variation in case rates across countries. Figure 2 shows that mean rates of full vaccination increased gradually across the study period, from 64.8% to 69.0%. The mean rates of booster vaccination started from a low level but increased quickly over time from 2.71% to 25.4%. Figure 3 shows that the average severity of public health restrictions (i.e., Stringency Index) across all countries increased gradually over time, from 41.3 to 50.1. Data on COVID-19 case rates were available for all countries. Over the entire study period and across all countries, data were missing for 212 observations for booster vaccination (27.6%), 130 observations for full vaccination (16.9%), and 66 observations for Stringency Index (8.6%).

The longitudinal Poisson regression analysis (see Table 1) found that, after controlling for country and time fixed effects, for every additional percent increase in full vaccination coverage, COVID-19 case rates decreased by a factor of 0.83 (or approximately 17%). This result was strongly statistically significant (*p* < 0.001). The observed 4.2% increase in mean vaccination rate across the study period corresponds with an IRR of 0.46 (0.83^4.2^), meaning that vaccines reduced case rates by 54% over the study period, when holding other variables constant. For every one-unit increase in Stringency Index, case rates decreased by a factor of 0.98, or approximately 2% (*p* = 0.019). An increase in booster vaccination coverage did lead to a significant decrease in case rates.

**TABLE 1:** Fixed effects Poisson regression model: the effect of vaccination on COVID-19 case rates^a^ (Europe, 13th October 2021–01st January 2022).

Variable	Rate ratio (RR) (95%CI)	*p*-Value
Population fully vaccinated (%)	0.829 (0.770–0.892)	<0.001
Population boosted (%)	1.015 (0.982–1.050)	0.368
Stringency Index	0.982 (0.968–0.997)	0.019

a Adjusted for country and time fixed effects.

Figure 2 shows the strength with which full vaccination predicted fewer COVID-19 case rates over time. The effect of vaccines on case rates remained steady until the detection of the Omicron variant in Europe, after which point the level of protection steadily diminished. The confidence intervals for all time periods overlapped and did not cross zero, meaning that an increase in full vaccination coverage provided some (albeit diminishing) protection against disease spread, up to approximately 5 weeks after the detection of Omicron in Europe. Figure 3 shows that booster vaccines did not reduce COVID-19 case rates at any point in time, with confidence intervals narrowing over time but including zero throughout.

## Discussion

A small increase in full vaccination coverage prevented many COVID-19 cases during the first 3 months of the fourth wave of infections across Europe. This protective effect decreased over time but remained significant up to 5 weeks after the Omicron variant was first detected in Europe. Booster vaccine coverage increased quickly but did not provide significant additional benefit for population disease control. The severity of public health restrictions increased slightly but had only a small impact on COVID-19 case rates.

### Impact of Vaccination

Recently published research has shown that vaccines were 79.6% (95% CI, 76.7–82.1) effective at preventing symptomatic disease with the Delta variant [22]. Case rates are also influenced by the effectiveness of vaccines in reducing transmission among those with breakthrough infections. A 2021 cohort study found that the durations of both infectious virus shedding and symptoms were significantly reduced in vaccinated individuals compared with unvaccinated individuals [23]. A Scottish study using data from 194,362 unvaccinated household members of 144,525 vaccinated health care workers found that the relative risk of COVID-19 infection was 0.46 (95% CI, 0.30–0.70) in household members, from 2 weeks after the second dose of vaccine in a healthcare worker [24]. This suggests that, for the Delta variant, vaccines provided an additional benefit in controlling disease, over and above individual protection against symptomatic disease.

Our real-world estimates show large decreases in case rates, for small increases in full vaccination. Previous studies comparing rates of disease between vaccinated and unvaccinated groups are typically unable to capture the population transmission or herd effects of vaccination, in addition to the benefit in preventing symptomatic disease. Any impact on community transmission may have played a particularly important role over the study period since transmission of respiratory illness is typically high during the late Autumn and Winter season.

Data on the effectiveness of vaccines against Omicron is limited. A recent UK test negative case-control pre-print study with 581 symptomatic Omicron cases found that full vaccination with the Oxford-AstraZeneca vaccine (ChAdOx1) did not protect against symptomatic disease, but full vaccination with the Pfizer (BNT162b2) vaccine provided a 34%–37% reduction in risk, after 15 weeks [12]. Vaccine effectiveness increased to 71.4% (95%CI: 41.8–86.0%) and 75.5% (95%CI: 56.1–86.3%) after a booster dose, for those who received a primary course of ChAdOx1 and BNT162b2, respectively. Here we demonstrate that despite a rapid increase in booster coverage across the European population, overall coverage remains relatively low, and they have not yet had a substantial impact upon disease control. We also found that full vaccination remained effective in reducing case rates up to 5 weeks after Omicron was detected in Europe. Although the average marginal effect decreased over time, and may continue to do so, a primary course of vaccines still offered a useful level of protection at the population level, during the initial phase of the response to a novel variant.

### Impact of Public Health Restrictions

Public health restrictions are also known to have some impact on disease control. The effectiveness of measures such as universal lockdowns and closures of businesses and schools for the containment of COVID-19 have largely been effective but depend on early implementation [25] and the pre-existing level of restrictions [26]. Despite an escalation in public health restrictions in some countries, we found that on average, more severe restrictions reduced case rates only slightly.

Over the study period, most European countries did not ban public events, close schools or businesses, or institute widespread lockdowns [27]. The main factor underpinning the moderate increase in Stringency Index across Europe was the additional travel restrictions put in place in response to Omicron [28]. The effectiveness of such restrictions in controlling the spread of COVID-19 is uncertain but thought to be limited [25]. The WHO currently advise against blanket travel bans, recommending that countries of departure, transit and arrival may apply a multi-layered risk mitigation approach to potentially delay and/or reduce the exportation or importation of any new variant [29]. Although the changes observed in Stringency Index here were not exclusively due to travel restrictions, the limited observed impact in our study may be largely due to the nature of the restrictions put in place, and the variability in the timing of restrictions across countries.

### Strengths and Limitations

We used a quasi-experimental methodology to evaluate the real-world impact of COVID-19 vaccines on disease control. Although previous epidemiological studies have investigated the impact of vaccines within a single population [30–36], by looking at multiple populations over time, we were able to evaluate the overall impact of vaccines across Europe, which may differ to their impact in any single population at one point in time. Our statistical analyses accounted for important differences across countries and over time. We were able to disentangle the impact of full vaccination from that of booster vaccination and public health restrictions, providing important insights for the ongoing COVID-19 response.

Our study has several limitations. First, we used COVID-19 case rates as our outcome measure and so cannot draw concrete conclusions about the real-world impact of vaccines on the Omicron variant specifically. Future research is required to understand how the real-world effectiveness of vaccination changes once Omicron is the dominant variant across Europe and more data is available. Second, although our study adjusts for most observed and unobserved confounders, there may be some time and country-variant confounders that we were not able to adjust for. One example may be compliance with facemask-wearing. This could be associated with recent changes in vaccination, as well as with COVID-19 case rates, and does not lie on the causal pathway between the two. Unfortunately, due to a lack of high-quality country-level data we were unable to separate this out from the impact of vaccination. By including the severity of public health restrictions as a time and country-variant confounder in our model, we hope to capture these effects at least partially. Third, there may have been COVID-19 cases with mild (or no) symptoms that were not detected through testing and recorded in national statistics, leading to ascertainment bias. This could mean our overall estimate of vaccine effectiveness is larger than the true value. However, we do not expect case ascertainment to be vastly different within countries over the relatively short study period, with data on test positivity (reflecting both COVID-19 prevalence and testing capacity) remaining similar for included countries with available data [37]. Fourth, vaccination data were missing for some countries. But given that we investigate impact within countries rather than between, and case rates were not systematically different in countries or time points for which there was missing data, this is unlikely to significantly bias the results. Finally, although fixed effects models are an often-used quasi-experimental method [38], inferences about causality can be less definitive than those obtained through a well-designed randomised controlled trial. But given the ubiquitous rollout of vaccines across Europe, it would not be possible to evaluate real-world impact in this way, making quasi-experimental methods the gold standard for vaccine programme evaluation.

### Implications

There is no international standard on disease control for COVID-19, with countries pursuing their own locally determined objectives [39]. Nevertheless, disease control is an important goal for all countries as it minimizes deaths, pressures on the health service, the economic impact of isolation and quarantine, and the risk of new variants emerging. Despite the limited efficacy of vaccines against symptomatic disease [22] and the waning of immunity over time [40], we show that vaccines were valuable in limiting case rates in Europe. Although they may become less useful as the Omicron variant continues to spread, they were beneficial up to 5 weeks after the detection of Omicron in Europe. Full vaccination may therefore be a core element of preparedness for future novel variants, even though at the individual-level the novel variant may be less susceptible to existing vaccines. This is because at a population-level, vaccines will provide some protection in the initial phase while a novel variant is not yet dominant, and can give countries additional time to scale up other disease control and mitigation measures, whilst limiting sudden pressure on health services. The fact that public health restrictions had only a slight impact on case rates further highlights the importance of vaccines. Policymakers must be careful of overreliance on restrictive government responses to control rates of disease, especially when very severe population-wide restrictions (such as lockdowns) are not a feasible or desirable option.

The WHO have advised that the rush for booster doses in response to the Omicron variant is likely to perpetuate existing vaccine inequity, prolong the pandemic and risk the emergence of new variants [41]. The distribution of vaccines across countries during the pandemic has been highly inequitable [42], with access largely along the lines of national wealth [42]. Our findings, from a region with a high level of access to booster doses, suggest that it will take several weeks before booster coverage is at a high enough level to benefit pandemic control. There may be other important reasons to prioritize booster doses such as to limit hospitalizations and deaths, but the global rush on boosters in response to the Omicron variant could derail important public health efforts to scale up full vaccination coverage. Given the limited increase in full vaccine coverage over the study period addressing hesitancy and opposition to vaccines, which has been an issue in several European populations [43], is vitally needed for disease control both now and in the future.

## Data Availability

All open-access data used in the analysis will be made available to researchers upon reasonable request to the corresponding author.

## Supplementary Material

Supplementary File S1 Histograms for included variables (Europe, 13th October 2021–01st January 2022).
