# Supplementary material for: The Real-World Impact of Vaccination on COVID-19 Cases During Europe’s Fourth Wave
Source: Int J Public Health. 2022 Jul 5;67:1604793. doi: 10.3389/ijph.2022.1604793 (PMC9294143; doi:10.3389/ijph.2022.1604793)
Supplement: Supplementary file 1 [file DataSheet1.docx]

**Supplementary File**

Histograms for observed variables included in models
